# Supplementary material for: Disentangling the critical signatures of neural activity
Source: Sci Rep. 2022 Jun 24;12:10770. doi: 10.1038/s41598-022-13686-0 (PMC9232560; doi:10.1038/s41598-022-13686-0)
Supplement: Supplementary file 1 — Supplementary Information. [file 41598_2022_13686_MOESM1_ESM.pdf]

Supplementary Information: Disentangling the critical signatures of neural activity

Benedetta Mariani,[1,2] Giorgio Nicoletti,[1,2] Marta Bisio,[2,3]
Marta Maschietto,[3] Stefano Vassanelli,[2,3] and Samir Suweis[1,2]

[1] *Laboratory of Interdisciplinary Physics, Department of Physics and Astronomy "G. Galilei", University of Padova, Padova, Italy*
[2] *Padova Neuroscience Center, University of Padova, Padova, Italy*
[3] *Department of Biomedical Science, University of Padova, Padova, Italy*

ADDITIONAL AVALANCHES STATISTICS IN THE MODEL

Another signature of criticality is the collapse of the average profile of avalanches of widely varying duration onto a single scaling function. For avalanches of duration T we can write down the average number of firing at time t as $s(t,T) = T^{\delta-1}F(t/T)$ where F is a universal scaling function that determines the shape of the average temporal profile. $\langle S(T)\rangle$ and $s(t,T)$ are related by $\langle S(T)\rangle = \int_0^T s(t,T)dt$. At the critical point we expect that plots of t/T versus $s(t,T)T^{1-\delta}$ for different T will collapse onto the same universal scaling function [1].

Thus, finding the exponent for which the goodness of the collapse is higher provides another way to estimate δ. For testing the avalanche shape collapse, we used the methodology introduced in [2]. To determine the quality of the collapse, the averaged and rescaled avalanche profiles of different lifetimes $F(t/T) = T^{1-\delta}s(t/T,T)$ are first linearly interpolated at 1000 points along the scaled duration. The variance across the different $F(t/T)$ is calculated at each interpolated point, and the shape collapse error $\epsilon(\delta)$ is then defined as the mean variance divided by the squared span of the avalanche shapes, where the span equals the maximum minus the minimum value of all rescaled avalanche profiles. In the presented analysis, avalanche shapes of $T > 10$ bins with at least 10 samples were used.

The collapse has been tested on the extrinsic model in the case of low $\mathcal{D}^*$, and the results are plotted in Figure S1. We find that the exponent that minimizes $\epsilon(\gamma)$ turns out to be ≈ 1.33, close to the estimates of δ found through the linear fit of average size given duration and through the prediction of the crackling-noise relation in the main text. Again, it is also close to the apparently super-universal exponent found in [3] and in [4].

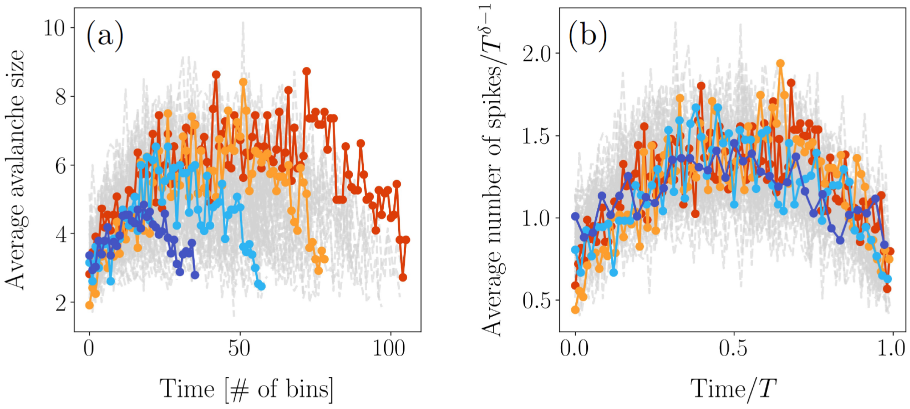

FIG. S1. Collapse of the average profile of avalanches of varying duration in the extrinsic model, for the low $\mathcal{D}^*$ regime. (a) Profile of the avalanches before the rescaling. (b) If we rescale with an exponent $\delta \approx 1.33$, which is remarkably close to the one found in the main text through the crackling-noise relation, we obtain an optimal collapse onto the same scaling function.

TESTING THE PREDICTIONS OF THE EXTRINSIC MODEL

Here, we show that simulations of the extrinsic model described by an Ornstein-Uhlenbeck process agree with the analytical results presented in the main text. Whenever not specified, we assume that the parameters of the model are given by $\mathcal{D}^* = 0.3$, $\theta = 1$, $\gamma_D = 10$ together with $\gamma_i = 0.1$, $\gamma_j = 0.5$. Thus, we are in the limit of timescale separation considered in the main text.

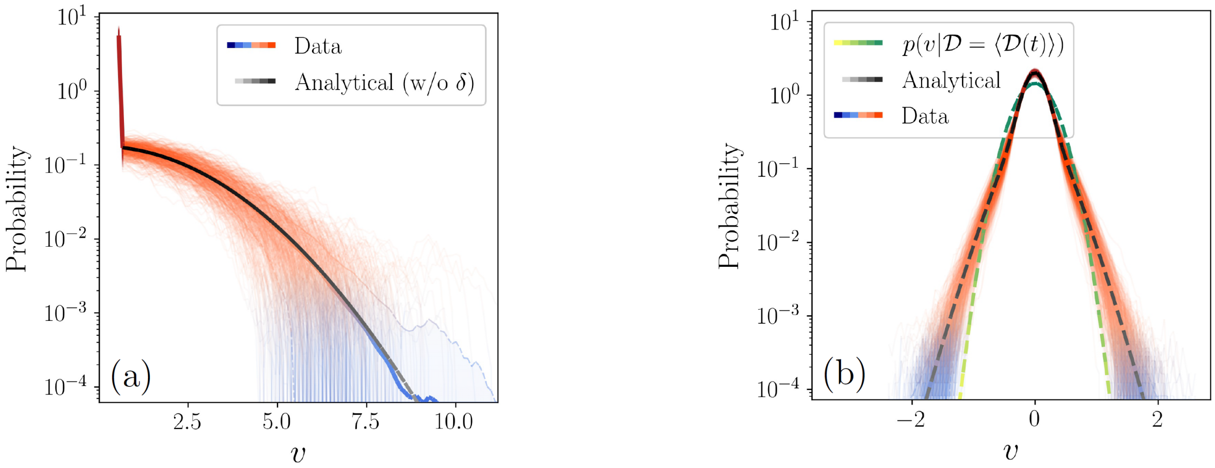

FIG. S2. Comparison between the stationary distributions obtained in the main text and the results 10^3 simulations. Semi-transparent lines represent different simulations. Filled areas of the plots represent one standard deviation from the mean distribution. (a) Probability distribution of $\mathcal{D}$. (b) Probability distribution of a single v_i.

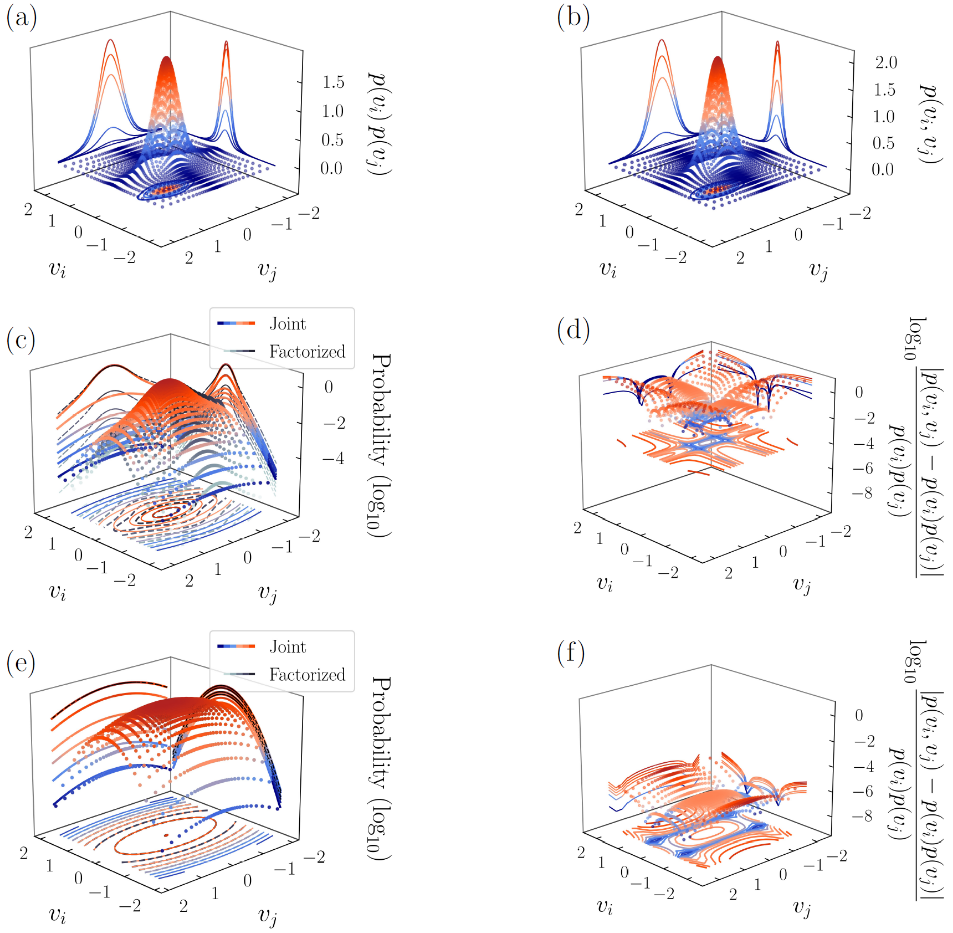

FIG. S3. Comparison between the analytical expressions of the joint probability distribution in Eq. (S1) and its factorization $p(v_i, v_j)$. (a) The factorized distribution. (b) The joint distribution. (c) Comparison between the two in a log-plot. (d) Relative difference between the two with respect to the factorized distribution. (e-f) Comparison between the joint and the factorized distributions, with the same parameters except for $\mathcal{D}^* = 5$.

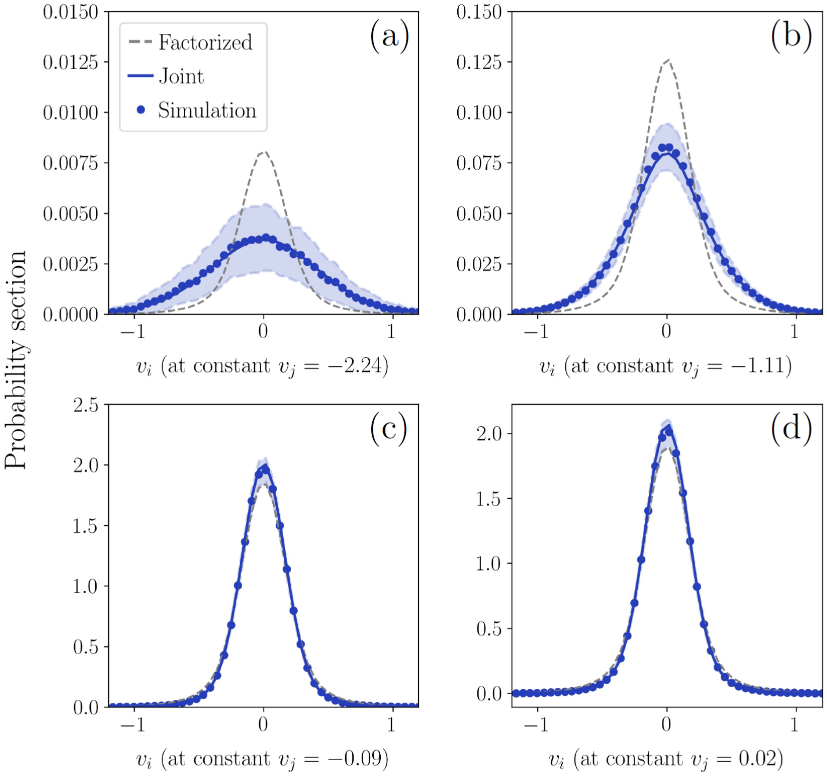

FIG. S4. Comparison between the analytical expressions of the joint probability distribution $p(v_i, v_j)$ given by Eq. (S1) and the one obtained from with 10^3 simulations. We also show the corresponding results for the factorized probabilities. The blue line corresponds to the analytical expression of $p(v_i, v_j)$. The corresponding dots represent the histogram of the distribution obtained from 10^3 simulations, and the semitransparent filled areas represent one standard deviation from this estimate. Similarly, the gray dashed line represent the analytical expression of $p(v_i)p(v_j)$. (a) Section along the v_j direction for small v_j, so that we are looking at the tails of the distribution. Even though the estimate along the tails is noisy, we clearly see that the estimate from the simulations lies along the analytical prediction. (b) As before, but for higher v_j. (c-d) As before, but with values of v_j close to zero so we look at the bulk of the distribution near its peak. Even though joint probability and its factorization now are more similar, once more the estimate from the simulation match the analytical expression $p(v_i, v_j)$.

Let us recall that

$$p(v_i, v_j) = \frac{1 + \mathrm{Erf}\left(\frac{\mathcal{D}^*}{\sqrt{\theta \gamma_D}}\right)}{2\pi \mathcal{D}^* \sqrt{\gamma_i \gamma_j}} e^{-\frac{1}{\mathcal{D}^*}\left(\frac{v_i^2}{\gamma_i} + \frac{v_j^2}{\gamma_i}\right)} + \frac{1}{\sqrt{\gamma_i \gamma_j \gamma_D \pi^3 \theta}} \int_{\mathcal{D}^*}^{\infty} \frac{dD}{D} e^{-\frac{1}{D}\left(\frac{v_i^2}{\gamma_i} + \frac{v_j^2}{\gamma_i}\right)} e^{-\frac{D^2}{\theta \gamma_D}} \tag{S1}$$

is the stationary joint probability of the model. In Figure S2a we check that the stationary distribution of $\mathcal{D}$ is indeed the one presented in the main text and in Figure S2b that the stationary distribution of a single v_i corresponds to the analytical expression we derived. If we compare this distribution to a standard distribution of an Ornstein-Uhlenbeck process with a diffusion coefficient equal to the mean $\langle \mathcal{D}(t) \rangle$ we immediately see that the distribution of our model is considerably more peaked around zero and displays longer tails. Indeed, one expects that due to the fact that $\mathcal{D}^* < \langle \mathcal{D}(t) \rangle$ the system tends to wander more easily close to zero, especially in the time windows where the diffusion coefficient is constant and equal to $\mathcal{D}^*$. At the same time, the fact that $\mathcal{D}(t)$ can change in time favors the presence of values of v that are larger in absolute value, which is the mechanism at the origin of the bursty behavior seen in the main text.

In Figure S3 we look instead at the properties of the joint probability distribution $p(v_i, v_j)$. The most natural quantity to compare this distribution with is its factorization $p(v_i)p(v_j)$, which is equivalent to ignoring the feedback effects between v_i and v_j due to the shared extrinsic modulation of $\mathcal{D}(t)$. Since we are setting $\mathcal{D}^* = 0.5$, we expect that these effects are going to be particularly relevant for the dynamics of the model. In particular, in Figure S3c-d we see that the most important differences between the two occur in the tails of the two-dimensional distribution, with the joint distribution typically showing dramatically longer tails. This translates to the fact that far-from-zero values of the two variables can occur more easily at the same time. The situation is completely reversed when we increase $\mathcal{D}^*$. In Figure S3e-f we see that for $\mathcal{D}^* = 8$ the joint probability distribution and the factorized distribution

are almost indistinguishable. Hence, this example shows explicitly that if $\mathcal{D}^*$ is high enough the dependence induced by the extrinsic modulation vanishes.

Let us keep focusing on the case $\mathcal{D}^* = 0.5$ for the time being. In Figure S4a-d we compare the one-dimensional sections of the analytical expression of the joint probability distribution with the results of 10^3 simulations of the model, together with the sections of the factorized distribution. The joint distribution estimated from the simulation matches particularly well the analytical prediction. Once more, and perhaps more clearly, in Figure S4a-b we see the stark difference that emerges along the tails between the joint probability distribution and its factorization. Interestingly, panel (c) and panel (d) show that the situation in the bulk of the distribution is reversed with respect to the tails and now the joint probability distribution is more peaked with respect to is factorization, albeit only slightly. That is, the modulation in the low $\mathcal{D}^*$ regime favors both large values of v_i and v_j and values very close to zero.

Overall, this brief analysis showed us how the bursty behavior that we see for small values of $\mathcal{D}^*$ emerges from the underlying probability distributions, which in turn emerge from the simple marginalization that occurs in Eq. (S1). Similar arguments, albeit impractical, could be carried out for the probability distributions beyond the two-point ones. In a sense, one could argue that the fundamental properties of the model are inherited from the fact that there are some unobserved physical quantities, and these are the quantities that drive the global response of the single variables.

For the specific case of a double Ornstein-Uhlenbeck process we chose, the net effect of the marginalization is the widening of the tails of both the one-point $p(v)$ and the two-point $p(v_i, v_j)$ probability distributions when $\mathcal{D}^*$ is small enough. As we increase $\mathcal{D}^*$, this effect becomes less and less important until it is completely negligible. In this sense, we can effectively think of $\mathcal{D}^*$ as a control parameter that changes the qualitative behavior of the system. Most importantly, the fact that the tails of the joint probability distribution are wider when $\mathcal{D}^*$ is small reflects dynamically in the emergence of a non trivial coordination between the variables, from which in turn power-law avalanches emerge.

CORRELATIONS IN THE INTERACTING MODEL

In Figure S5 we compare the correlation matrix obtained from the data, the interaction matrix $\tilde{A}_{ij}$ obtained by solving the inverse problem described in the main text, and the correlation matrix obtained from simulations of the resulting model.

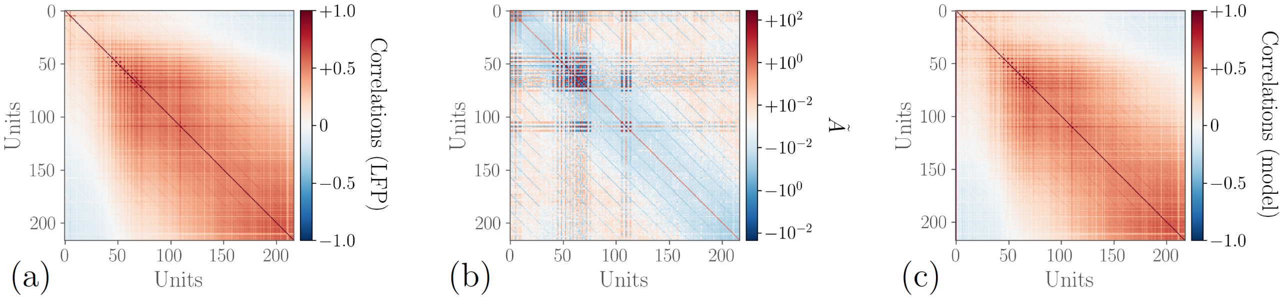

FIG. S5. Comparison between (a) the correlations of the data and (c) the correlations of the interacting model after solving the inverse problem. Panel (b) shows the inferred matrix $\tilde{A}$, which is independent on the parameters of the model.

AVALANCHES IN THE EXTRINSIC MODEL, DEPENDENCE ON $\mathcal{D}^*$ AND FINITE-SIZE SCALING

Figure S6 shows avalanches' distributions in the extrinsic model as $\mathcal{D}^*$ is increased. It is possible to see that, while the exponents converge to a fixed value for $\mathcal{D}^*$ sufficiently low, as $\mathcal{D}^*$ is increased they gradually increase in absolute value, and the distribution tends to an exponential when $\mathcal{D}^* > 5$. That is, the shift from exponential and power-law avalanches is smooth.

Figure S7 shows instead the finite-size scaling behavior of avalanches in the extrinsic model. The avalanches obtained do not present a clear cutoff. Yet, it is evident that when increasing the number of units the values of the maximum avalanches size and duration increase, while the exponent of the distribution remains the same, displaying a property typical of true power laws. This is perhaps unsurprising, since the units are conditionally independent to begin with. In [5] (SI) we have studied the avalanche finite-size scaling in LFPs from the same set of experiments, and they indeed reproduce the expected finite-size scaling features, as already found in the literature [6].

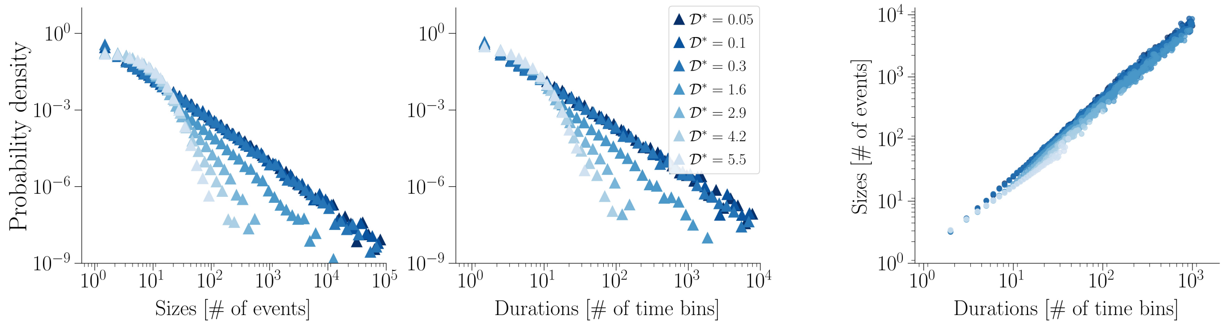

FIG. S6. Avalanches in the extrinsic model at increasing values of $\mathcal{D}^*$.

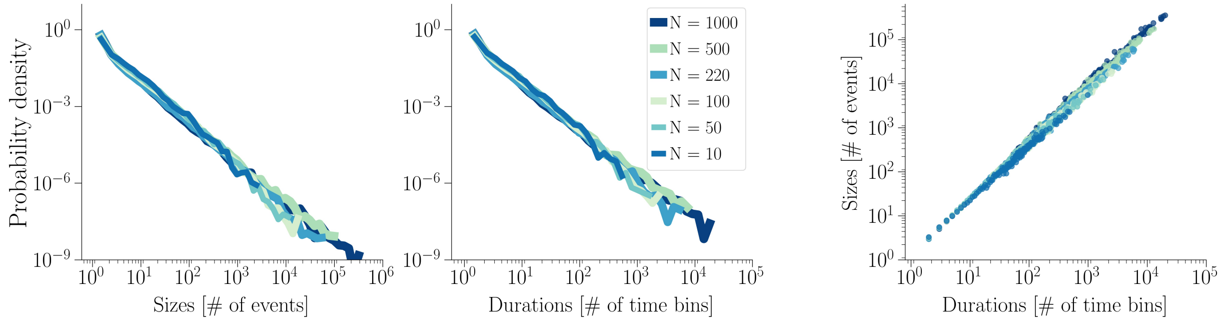

FIG. S7. Finite-size scaling in avalanches found in the extrinsic model.

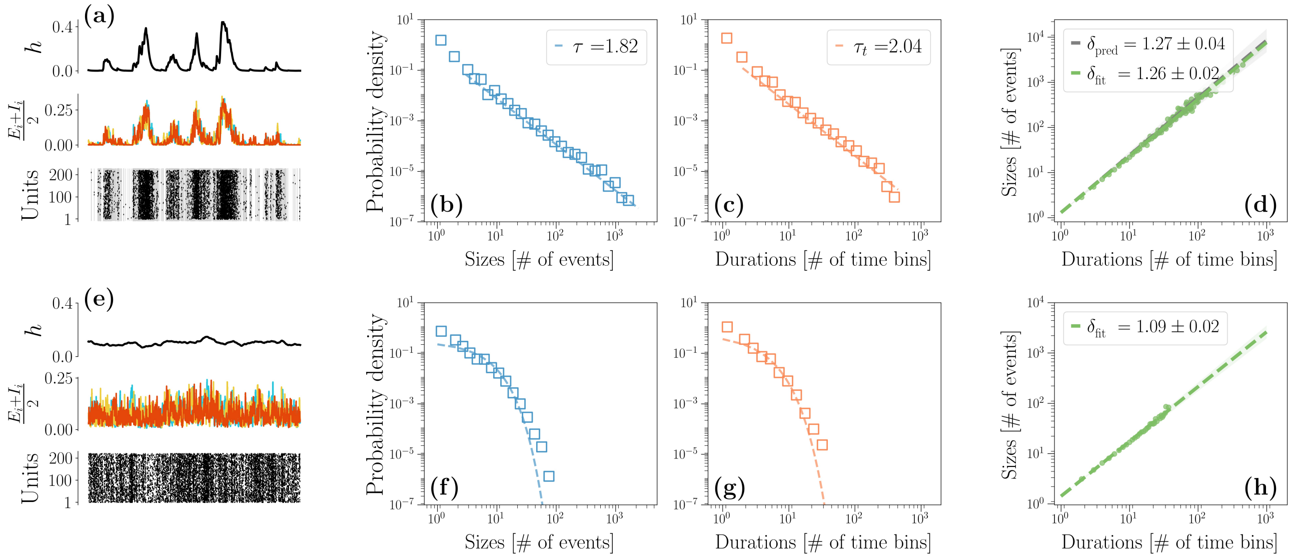

FIG. S8. Avalanche statistics generated by the Wilson-Cowan units. The "internal" Wilson-Cowan units are always in an inhibition dominated phase, i. e. $\omega_I = 7$ and $\omega_E = 6.8$, and $\alpha = 1$. Their external input h is instead always in a critical state, in particular $\omega_E^{(h)} = 50.05$, $\omega_I^{(h)} = 49.95$ and $\alpha^{(h)} = 0.1$, with $h^{(h)} = 10^{-3}$. In panels (a-d) The amplitude of the noise, is increased to 1.2×10^{-3}. In panels (e-h) instead the noise is reduced to 5×10^{-5}. In panels (a) and panel (e) we compare the trajectories of h, $\frac{E_i+I_i}{2}$ and the corresponding trains of events in the high (a) and low (e) $\sigma^{(h)}$ regime. (b-d) If $\sigma^{(h)}$ is high avalanches are power-law distributed and the crackling-noise relation is verified. (f-g) Same plots, now in the low $\sigma^{(h)}$ regime. Avalanches are now fitted with an exponential distribution. (h) The average avalanche size as a function of the duration scales with an exponent that, as $\sigma^{(h)}$ decreases, becomes closer to the trivial one $\delta_{\rm fit} \approx 1$.

A CRITICAL STOCHASTIC WILSON-COWAN MODEL AS EXTERNAL MODULATION

In Figure S8 we analyze some Wilson-Cowan units that receive the same input by another Wilson-Cowan population that is in a critical state. Indeed, in this case we set $\alpha^{(h)} = \omega_0^{(h)} = \omega_E^{(h)} - \omega_I^{(h)} = 0.1$, and we are exactly at the critical point of the stochastic Wilson-Cowan model as shown in [7]. Here, avalanches in principle will be present for all noise amplitudes (i.e., system sizes). However note that in Figure S8 we set $h^{(h)} = 10^{-3}$, so that the dynamics slightly deviates from the critical point defined for $h = 0$. For this reason, avalanches disappear if we reduce the noise to a sufficiently low value (see Figure S8e-h). Notably, the noise amplitude has to be dramatically reduced in order not to see avalanches, at $\sigma^{(h)} = 5 \times 10^{-5}$, corresponding to very large system sizes ($K = L \approx 4 \times 10^7$, with K and L respectively the number of excitatory and inhibitory neurons [8] corresponding to the neural population).

AVALANCHES STATISTICS ACROSS RATS

In Figure S9 we report the avalanches statistics from the other rats that we analyzed. The avalanches statistics is computed by considering all available the 20 trials of basal activity for each rat, that are 7.22s long. Inter-rat variability is present with respect to avalanche exponents, and is expected as found in previous experiments [3, 9, 10]. Moreover, a theoretical explanation for the difference in avalanche exponents has been also recently proposed as signature of quasi-criticality [11]. However, the fundamental point is that the crackling-noise relation is always verified compatibly with the experimental errors, a feature that is usually considered a hallmark of criticality [3].

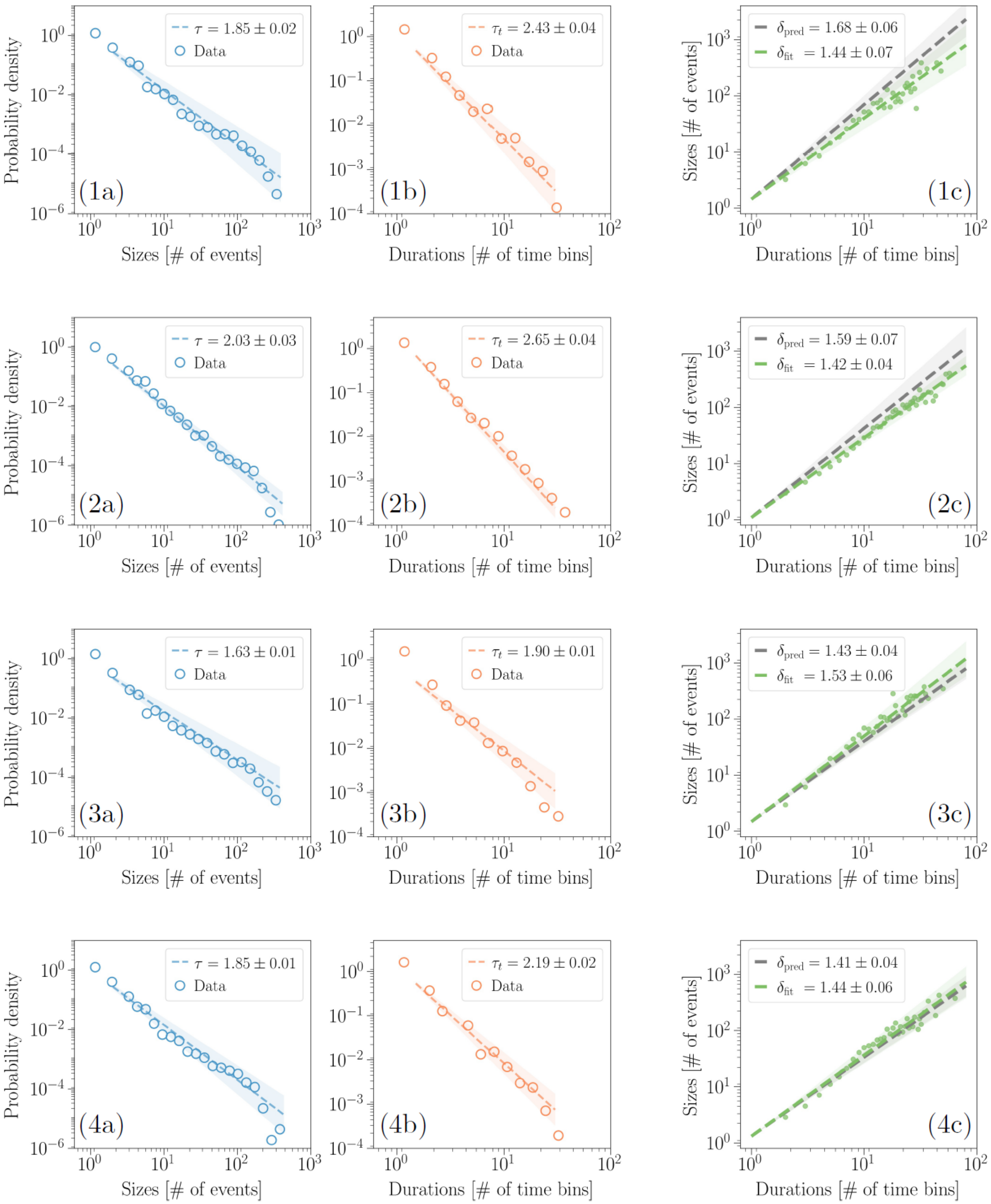

FIG. S9. Avalanche statistics in four different rats. (1a-4a) The distribution of the avalanches' sizes is consistently a power-law, with an exponent that slightly depends on the single rat. (1b-4b) The avalanche durations are once more power-law distributed in all rats with some variability in the exponents, even though the range accessible with the experimental setup only covers two decades. (1c-4c) The crackling-noise relation, however, is consistently satisfied in each rat.

[1] J. Sethna, K. Dahmen., and C. Myers, Crackling noise, Nature **410**, 242–250 (2001).

[2] N. Marshall, N. M. Timme, N. Bennett, M. Ripp, E. Lautzenhiser, and J. M. Beggs, Analysis of power laws, shape collapses, and neural complexity: New techniques and matlab support via the ncc toolbox, Frontiers in Physiology **7**, 250 (2016).

[3] A. J. Fontenele, N. A. P. de Vasconcelos, T. Feliciano, L. A. A. Aguiar, C. Soares-Cunha, B. Coimbra, L. Dalla Porta, S. Ribeiro, A. J. a. Rodrigues, N. Sousa, P. V. Carelli, and M. Copelli, Criticality between cortical states, Physical Review Letters **122**, 208101 (2019).

[4] N. Friedman, S. Ito, B. A. W. Brinkman, M. Shimono, R. E. L. DeVille, K. A. Dahmen, J. M. Beggs, and T. C. Butler, Universal critical dynamics in high resolution neuronal avalanche data, Physical Review Letters **108**, 208102 (2012).

[5] B. Mariani, G. Nicoletti, M. Bisio, M. Maschietto, R. Oboe, A. Leparulo, S. Suweis, and S. Vassanelli, Neuronal avalanches across the rat somatosensory barrel cortex and the effect of single whisker stimulation, Frontiers in Systems Neuroscience **15**, 89 (2021).

[6] J. M. Beggs and D. Plenz., Neuronal avalanches in neocortical circuits, Journal of Neuroscience **12**, 23(35):11167–11177 (2003).

[7] A. de Candia, A. Sarracino, I. Apicella, and L. de Arcangelis, Critical behaviour of the stochastic wilson-cowan model, PLOS Computational Biology **17**, 1 (2021).

[8] M. Benayoun, J. D. Cowan, W. van Drongelen, and E. Wallace, Avalanches in a stochastic model of spiking neurons, PLOS Computational Biology **6**, 1 (2010).

[9] W. Shew, W. Clawson, J. Pobst, and al, Adaptation to sensory input tunes visual cortex to criticality., Nature Phys. **11**, 659–663 (2015).

[10] K. Bansal, J. O. Garcia, N. Lauharatanahirun, S. F. Muldoon, P. Sajda, and J. M. Vettel, Scale-specific dynamics of large-amplitude bursts in eeg capture behaviorally meaningful variability (2020), arXiv:2003.06463 [q-bio.NC].

[11] L. J. Fosque, R. V. Williams-García, J. M. Beggs, and G. Ortiz, Evidence for Quasicritical Brain Dynamics, Physical Review Letters **126**, 098101 (2021), 2010.02938.
